# 1874. Comprehensive, longitudinal wastewater surveillance for SARS-CoV-2 across a university campus, demonstrates low levels of SARS-CoV-2 activity relative to the surrounding community

**DOI:** 10.1093/ofid/ofac492.1501

**Published:** 2022-12-15

**Authors:** Jangwoo Lee, Nicole Acosta, Jennifer Van Doorn, Kashtin Low, Paul Westlund, Maria Bautista Chavarriaga, Barbara Jean M. Waddell, Kristine Du, Janine McCalder, Puja Pradhan, Navid Sedaghat, Chloe Papparis, Jianwei Chen, Kevin Xiang, Leslie Chan, Laura Vivas, Norma J Ruecker, Brendan Webster, Jon Meddings, Gopal Achari, M Cathryn Ryan, Rhonda Clark, Kevin Frankowski, Casey R J Hubert, Michael Parkins

**Affiliations:** University of Calgary, Calgary, Alberta, Canada; University of Calgary, Calgary, Alberta, Canada; University of Calgary, Calgary, Alberta, Canada; University of Calgary, Calgary, Alberta, Canada; C.E.C. Analytics, Calgary, Alberta, Canada; University of Calgary, Calgary, Alberta, Canada; University of Calgary, Calgary, Alberta, Canada; University of Calgary, Calgary, Alberta, Canada; University of Calgary, Calgary, Alberta, Canada; University of Calgary, Calgary, Alberta, Canada; University of Calgary, Calgary, Alberta, Canada; University of Calgary, Calgary, Alberta, Canada; University of Calgary, Calgary, Alberta, Canada; University of Calgary, Calgary, Alberta, Canada; University of Calgary, Calgary, Alberta, Canada; University of Calgary, Calgary, Alberta, Canada; Calgary City, Calgary, Alberta, Canada; University of Calgary, Calgary, Alberta, Canada; University of Calgary, Calgary, Alberta, Canada; University of Calgary, Calgary, Alberta, Canada; University of Calgary, Calgary, Alberta, Canada; University of Calgary, Calgary, Alberta, Canada; University of Calgary, Calgary, Alberta, Canada; University of Calgary, Calgary, Alberta, Canada; University of Calgary, Calgary, Alberta, Canada

## Abstract

**Background:**

Universities are interactive communities where frequent contacts between individuals occur, increasing the risk of outbreaks of COVID-19. We embarked upon a real-time wastewater (WW) monitoring program across the University of Calgary (UofC) campus measuring WW SARS-CoV-2 burden relative to levels of disease in the broader surrounding community.

**Figure 1:**
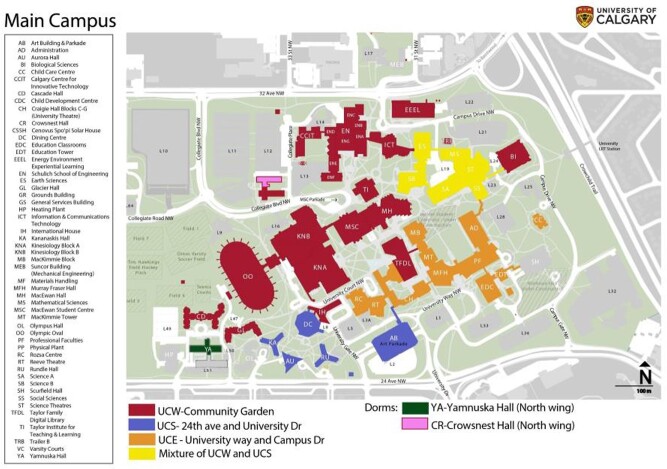

The colour scheme shows 6 sewer sub-catchments at the University of Calgary. Autosamplers were deployed at 4 sampling nodes within sub-catchments CR and YA (both residence halls), and UCE and UCS (catchments that include several campus buildings).

**Figure 2:**
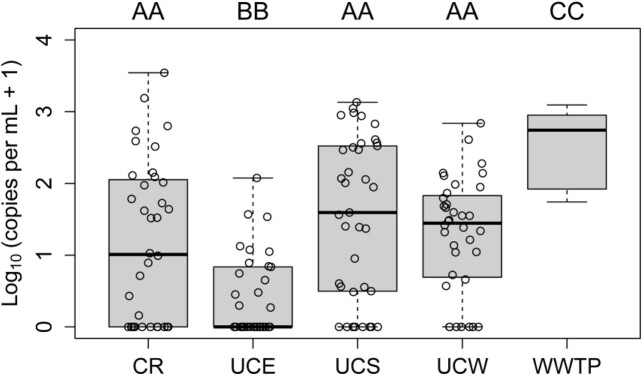

Log10-transformed abundance (i.e., copies per mL) of nucleocapsid gene (i.e., N1) for SARS-CoV-2 for each sampling location during October 2021 – April 2022. Locations denoted by the same letters (A, B, or C) show no statistical difference (p > 0.05) according to the Wilcoxon rank-sum test. The WWTP sample corresponds to a catchment area covering most of Calgary including the university campus, for which sampling locations CR, UCE, UCS, and UCW are defined in Fig. 1.

**Methods:**

From October 2021 – April 2022, WW was collected thrice weekly across UofC campus through 4 individual sewer sampling nodes (Fig. 1) using autosamplers (C.E.C. Analytics, CA). Results from these 4 nodes were compared with community monitoring at Calgary’s largest WW treatment plant (WWTP), which received WW from surrounding neighborhoods, and also from UofC. Nucleic acid was extracted from WW for RTqPCR quantification of the N1 nucleocapside gene from SARS-CoV-2 genomic RNA. Qualitative (positive samples defined if cycle threshold < 40) and quantitative statistical analyses were performed using R.

**Results:**

Levels of SARS-CoV-2 in WW were significantly lower at all campus monitoring sites relative to the WWTP (Wilcoxon rank-sum test p < 0.05; Fig. 2). The proportion of WW samples that were positive for SARS-CoV-2 was significantly higher for WWTP than at least two campus locations (p < 0.05 for Crowsnest Hall and UCE - University way and campus drive) according to Fischer’s exact 2-sided test. The proportion of WW samples with positive WW signals were still higher for WWTP than the other two locations, but statistically not significant (p = 0.216). Among campus locations, the buildings in UCE catchment showed much lower N1 signals than other catchments, likely owing to buildings in this catchment primarily being administration and classroom environments, with lower human-to-human contact and less defecation compared to the other 3 catchments, which include residence hall, a dining area, and/or laboratory spaces.

**Conclusion:**

Our results show that SARS-CoV-2 RNA shedding in WW at the UofC is significantly lower than the city-wide signal associated with surrounding neighborhoods. Furthermore, we demonstrate that WW testing at well-defined nodes is a sampling strategy for potentially locating specific places where high transmission of infectious disease occurs.

**Disclosures:**

**All Authors**: No reported disclosures.

